# The Current Landscape of Oncolytic Herpes Simplex Viruses as Novel Therapies for Brain Malignancies

**DOI:** 10.3390/v13061158

**Published:** 2021-06-17

**Authors:** Joshua D. Bernstock, Samantha E. Hoffman, Jason A. Chen, Saksham Gupta, Ari D. Kappel, Timothy R. Smith, E. Antonio Chiocca

**Affiliations:** 1 Department of Neurosurgery, Brigham and Women’s Hospital, Harvard Medical School, Boston, MA 02115, USA; jbernstock@partners.org (J.D.B.); shoffman@hms.harvard.edu (S.E.H.); jchen118@mgh.harvard.edu (J.A.C.); sgupta@bwh.harvard.edu (S.G.); akappel@bwh.harvard.edu (A.D.K.); trsmith@partners.org (T.R.S.); 2 Harvard-MIT MD-PhD Program, Harvard Medical School, Boston, MA 02115, USA; 3 Computational Neuroscience Outcomes Center, Brigham & Women’s Hospital, Harvard Medical School, Boston, MA 02115, USA

**Keywords:** oncolytic virus, herpes simplex virus (HSV), immunotherapy, immunovirotherapy, glioblastoma (GBM), brain tumors

## Abstract

Despite advances in surgical resection and chemoradiation, high-grade brain tumors continue to be associated with significant morbidity/mortality. Novel therapeutic strategies and approaches are, therefore, desperately needed for patients and their families. Given the success experienced in treating multiple other forms of cancer, immunotherapy and, in particular, immunovirotherapy are at the forefront amongst novel therapeutic strategies that are currently under investigation for incurable brain tumors. Accordingly, herein, we provide a focused mini review of pertinent oncolytic herpes viruses (oHSV) that are being investigated in clinical trials.

## 1. Introduction

Central nervous system (CNS) malignancies comprise one of the major causes of cancer-related morbidity and mortality in both adult and pediatric populations [1]. Despite advancements in radio- and chemotherapeutic regimens, malignant CNS neoplasms arguably result in the most dismal prognoses of any tumor type. Existing therapeutic options may also contribute to additional neurological morbidities such as cognitive impairment, psychological disturbance, and, in severe cases, seizures and paralysis [2]. These effects are particularly pronounced in pediatric populations [3]. 

The successes of immunotherapy in other solid tumors have spurred interest in leveraging similar treatments to control brain malignancies. Of the immunotherapy modalities currently undergoing clinical trials, oncolytic herpes simplex viruses (oHSVs) have emerged as leading contenders due to several key advantages. First, oHSVs leverage the inherent neurotropism of HSV, allowing the selective targeting of neural malignancies and, thus, decreasing the likelihood of off-target toxicities [4]. Damage to non-tumor cells is further minimized by deleting or controlling the γ134.5 gene in oHSVs, which is required for HSV replication in normal cells but not tumor cells [5]. Finally, oHSVs not only induce cell death in infected cells, but also recruit immune effectors to the tumor site; other immunotherapies may only produce the latter effect. In line with such thinking, mouse models of GBM T cell responses to both tumor and viral antigens correlate with therapeutic responses [6]. 

In this review, we summarize the landscape of oHSVs used in clinical trials for CNS malignancies and characterize the challenges and promises of this novel immunotherapeutic approach.

## 2. rQNestin 34.5v2

While deletion of γ134.5 protects non-malignant cells from oHSV replication, absence of the gene may also impair replicative potential in targeted cells. The rQNestin 34.5v2 oHSV includes both dual deletion of γ134.5 and a copy of γ134.5 under the control of the *nestin* promoter [7,8,9]. Nestin is highly expressed in gliomas but not in normal CNS-resident cells; the genetic design of rQNestin 34.5v2, thereby, restores viral replicative ability in infected tumor cells without rendering normal neurons/glia vulnerable to oHSV lysis [7,10].

Like other oHSVs, inoculation of rQNestin 34.5v2 induces both innate and adaptive immunity. Data from rat glioma models demonstrated an increase in tumor-infiltrating CD68+ macrophages and natural killer (NK) cells within 6 hours of rQNestin inoculation [11]. Increased interferon-gamma (IFN-γ) levels produced by these innate effectors suppressed viral replication; treatment with the immunomodulator cyclophosphamide relieved this inhibition and enhanced oncolysis. Of note, infiltration by helper CD4+ T cells and cytotoxic CD8+ T cells occurred significantly later and was augmented by cyclophosphamide treatment [11]. 

These data informed the design of the sole phase I clinical trial of rQNestin 34.5v2, which is currently underway (Table 1). One hundred and eight adult patients with recurrent or progressive malignant gliomas will receive intratumoral injection during biopsy surgery with or without preoperative cyclophosphamide. The results of this study will elucidate the safety and efficacy of rQNestin 34.5v2 in human high-grade glioma, as well as the impact of innate immune suppression by cyclophosphamide on viral therapy.

## 3. HSV G207 and M032 HSV-1

G207 not only includes deletions of both copies of γ134.5, but also harbors a lacZ operon insertion into the HSV-1 U_L_39 (*ICP6*) gene [12]. The latter alteration disrupts viral ribonucleotide reductase activity, inhibiting HSV DNA synthesis, which further inhibits oHSV replication in normal cells. Given retention of the thymidine kinase (*TK*) gene, these mutations also confer hypersensitivity to ganciclovir and acyclovir, allowing rapid inhibition of viral therapy should adverse events occur. Similar to other oHSVs, G207 promotes brain tumor death by both direct oncolysis and recruitment of cytotoxic tumor-infiltrating immune effectors. Modifying G207 via the insertion of IL-12, a pro-inflammatory cytokine, can further augment the adaptive immune response to oHSV inoculation. IL-12 has a mitogenic effect on cytotoxic T cells and NK cells and has anti-angiogenic properties; its potent anti-tumoral capacity has been demonstrated in a variety of human cancer types [21]. Inoculation with M032-HSV-1, a R3659-IL12 construct [22], was shown to be safe and efficacious in non-human glioma models; a phase I clinical trial of M032 in recurrent malignant gliomas is ongoing (Table 1) [20,23].

The majority of past oHSV clinical trials for patients with brain tumors employed the HSV G207 virus (Table 1). To date, three adult phase I clinical trials have been completed, all of which involved G207 inoculation of recurrent high-grade gliomas in adult patients [12,15,16]. Radiographic response was observed in all three studies, and no dose-limiting toxicities were identified, highlighting the safety and efficacy of G207 as a vector for therapy. Importantly, no viral shedding was detected in any patient; critically, previous exposure to wildtype HSV-1 did not impair response to oHSV therapy [24].

Interestingly, work has recently emerged to suggest that pediatric brain tumors are even more sensitive to oHSVs then adult tumors [25], and as such, G207 is also under investigation as a potential therapy for pediatric CNS malignancies, which are the leading cause of cancer-related mortality in patients aged 0–19 years old [26]. Unlike adult brain tumors, which are usually supratentorial, pediatric brain tumors most commonly occur in infratentorial structures and may, therefore, be less amenable to safe surgical resection. Cerebellar G207 inoculation into murine brains was both non-toxic and efficacious against medulloblastoma xenografts, suggesting that G207 may be a potent vector for treating aggressive pediatric malignancies [27]. One clinical trial of G207 in recurrent pediatric cerebellar brain tumors is currently recruiting patients [3]; another phase I study focusing on G207’s safety profile as a treatment for pediatric high-grade glioma has recently finished and has reported impressive results with radiographic, neuropathological, and/or clinical responses having been demonstrated in 11 out of 12 patients. Critically, the median overall survival was 12.2 months in those patients who received G207 vs. 5.6 months in historical controls [13] (Table 1).

## 4. HSV G47∆ and HSV G47∆-IL12

Tumor heterogeneity is a known contributor to therapeutic resistance in high-grade brain malignancies. Specifically, these tumors harbor populations of glioma stem cells (GSCs), which commonly evade elimination by conventional radiation and chemotherapeutic approaches [28,29,30,31]. GSCs have been implicated in tumorigenesis, proliferation, and tumor repopulation/recurrence following chemotherapy; eliminating this population is, therefore, crucial to producing a durable therapeutic response [32]. Prior attempts to target GSCs have been complicated by toxicity to neuronal stem cells, which bear similar molecular profiles and self-renewing capabilities [33]. The cancer-selective activity of oHSVs make them ideal candidates to target this elusive population, conferring another benefit over other immunotherapy options. 

In an attempt to address GSCs [34], G207 was further engineered to produce HSV G47**∆**, which harbors an additional deletion in the viral *ICP47* gene and the US11 promoter. G47**∆** facilitates elimination of GSCs through not only efficient killing of infected GSCs, but also reducing the self-renewal capacity of these tumorigenic precursors. Deletion of the US11 promoter increases the replicative potential of G47**∆**, compensating for the dual deletions in γ134.5, thereby augmenting oncolytic capacity [35]. Inoculation with G47**∆** also appears to produce a potent anti-angiogenic environment, a secondary mechanism by which G47**∆** may decrease the tumor burden [36].

With regard to its immunogenic potential, it should be noted that *ICP47* inhibits the transporter associated with antigen presentation, preventing antigen presentation by MHC class I molecules and, thus, CD8+ T cell activation and response [37]. Deletion of *ICP47* may, therefore, upregulate MHC I expression in G47**∆**-infected cells, promoting a cytotoxic immune response. Interestingly, the application of G47**∆** to human melanoma samples suggests increased stimulation of tumor-infiltrating lymphocytes [35]. Arming G47**∆** with the cytokine IL-12 both stimulates cytotoxic T cell activity and inhibits immunosuppressive regulatory T cells, conferring a survival advantage in murine models of glioblastoma [38]. The re-engineered G47**∆** virus had similar safety and efficacy as the unaltered G47**∆** strain in murine models; future clinical trials will facilitate a similar comparison in human brain tumors [38]. Although G47**∆** has yet to enter clinical trial for treatment of brain cancer in the United States, a first-in-human, clinical trial of this third-generation oHSV was recently completed at the University of Tokyo in Japan [39]. Thirteen patients with recurrent or residual glioblastoma underwent repeated dosing with G47**∆**, with the majority of patients receiving maintenance temozolomide (TMZ) chemotherapy [39]. Investigators reported a one-year survival rate of 92.3% with few adverse events, highlighting the promise of G47**∆** as an effective immunotherapy option for aggressive CNS malignancies. Of note, a recent meta-analysis demonstrated that the median overall survival in recurrent GBM regardless of treatment modality, of which there is no standard, was 6.5 months [40].

## 5. HSV 1716 and HSV C134

Other oHSVs investigated for efficacy against CNS malignancies include HSV 1716 and HSV C134, both of which harbor deletions of the viral γ134.5 gene. C134 differs from 1716 due to expression of HCMV IRS1 that complements the loss of γ134.5. [41,42]. Two phase I clinical trials of 1716 in high-grade glioma have been conducted to date. The adult trial revealed no clinical toxicities associated with 1716 therapy, and several patients experienced long-term survival of over one year post-inoculation [17,18,19]. Five patients displayed an immunologic response to the virus, with elevated anti-HSV IgG antibody titers. [18]. The pediatric trial was terminated in 2016, although a trial of 1716 in pediatric patients with non-CNS solid tumors found the virus safe and effective [43]. A phase I clinical trial for C134 inoculation in adult recurrent glioblastoma has been registered on clinicaltrials.gov (accessed on 16 March 2020) but is not yet recruiting (Table 1). 

## 6. Combination Therapy Approaches

Strong interest exists in potentiating existing brain tumor therapy regimens via combination with oHSV inoculation. Arming oHSV with other anticancer genes was shown to augment efficacy [24]. Previous studies have demonstrated that combining oncolytic viral and ionizing radiation therapies produces a synergistic anti-tumoral effect, for which several mechanisms may be responsible. Data suggest that irradiation increases oHSV replication and cytotoxicity by upregulating DNA-repair mediator GADD34 and tumor cell ribonucleotide reductase activity [44,45]. Finally, oHSV infection may interfere with DNA repair in irradiated cells, hastening the induction of apoptosis [46]. A phase I study of G207 inoculation followed by radiation in adult patients with recurrent, malignant glioma confirmed the potential of combination therapy to enhance clinical response in humans (Table 1) [15]. 

The efficacy of combining oHSV therapy with current chemotherapeutic regimens is an area of active investigation. In vitro studies combining either G207 or G47**∆** with TMZ, the standard-of-care chemotherapy for high-grade gliomas, demonstrated enhanced glioma lysis compared to viral therapy alone [47,48]. Similar to radiation, TMZ induces tumor cells upregulate GADD34 and ribonucleotide reductase, enhancing oHSV viral replication in infected cells [47]. Clinical trials combining oHSVs with TMZ and other chemotherapeutic agents will be crucial in determining the safety, efficacy, and potential toxicities of dual viral chemotherapy in high-grade glioma [49].

## 7. Future Directions

oHSVs promise to advance immunotherapeutic management of adult and pediatric high-grade gliomas. Continued investigation is required to overcome current technical and microenvironmental obstacles inhibiting virotherapy success (reviewed by Totsch et al. [24]) with future combinations focusing on immune checkpoint inhibitors and cancer vaccinations harboring great promise. Identification of optimal viral–pharmacologic and viral–radiologic regimens will augment the potential of oHSVs to treat aggressive, previously incurable CNS malignancies. Finally, it will be important to further clarify the clinical role of both armed viruses and those engineered to be less attenuated with regard to safety and efficacy as the field of immunovirotherapy continues to advance. 

## Figures and Tables

**Table 1:** Ongoing and past oHSV clinical trials in brain tumor patients registered on clinicaltrials.gov (accessed on 16 March 2020).

Tumor Type	Virus Type	Clinical Trial ID	Clinical Trial Phase	Pediatric or Adult	Status (as of 3/16/2020)	Reference
Recurrent GBM	HSV C134	NCT03657576	1	Adult	Active, not recruiting	N/A
Glioma, Astrocytoma, GBM (recurrent)	HSV G207	NCT00028158	1b/2	Adult	Completed	[12]
Cerebellar brain tumors (recurrent, refractory)	HSV G207	NCT03911388	1	Pediatric	Recruiting	[3]
Supratentorial brain tumors	HSV G207	NCT02457845	1	Pediatric	Active, not recruiting	[13,14]
Recurrent HGG	HSV G207	NCT04482933	2	Pediatric	Not yet recruiting	N/A
Malignant glioma	HSV G207	NCT00157703	1	Adult	Completed	[15,16]
HGG (refractory, recurrent)	HSV-1716	NCT02031965	1	Pediatric	Terminated	N/A
HGG	HSV-1716			Adult	Completed	[17,18,19]
Recurrent malignant glioma	M032-HSV-1	NCT02062827	1	Adult	Recruiting	[20]
Recurrent malignant glioma	rQNestin 34.5 v.2	NCT03152318	1	Adult	Recruiting	[7,9]

N/A = no associated publication.
